# Translational assessment of a DATA-functionalized FAP inhibitor with facile ^68^Ga-labeling at room temperature

**DOI:** 10.1007/s00259-023-06285-2

**Published:** 2023-06-07

**Authors:** Alondra Escudero-Castellanos, Jens Kurth, Surachet Imlimthan, Elena Menéndez, Eirinaios Pilatis, Euy Sung Moon, Tilman Läppchen, Hendrik Rathke, Sarah M. Schwarzenböck, Bernd J. Krause, Frank Rösch, Axel Rominger, Eleni Gourni

**Affiliations:** 1 Department of Nuclear Medicine, Inselspital, Bern University Hospital, University of Bern, Bern, Switzerland; 2 Department of Nuclear Medicine, Rostock University Medical Centre, Rostock, Germany; 3 Department of Chemistry—TRIGA site, Johannes Gutenberg—University of Mainz, Mainz, Germany

**Keywords:** CAF, FAP inhibitors, Tumor stroma, PET imaging

## Abstract

**Purpose:**

The present study aims at evaluating the preclinical and the clinical performance of [^68^Ga]Ga-DATA^5m.^SA.FAPi, which has the advantage to be labeled with gallium-68 at room temperature.

**Methods:**

[^68^Ga]Ga-DATA^5m^.SA.FAPi was assessed in vitro on FAP-expressing stromal cells, followed by biodistribution and in vivo imaging on prostate and glioblastoma xenografts. Moreover, the clinical assessment of [^68^Ga]Ga-DATA^5m^.SA.FAPi was conducted on six patients with prostate cancer, aiming on investigating, biodistribution, biokinetics, and determining tumor uptake.

**Results:**

[^68^Ga]Ga-DATA^5m^.SA.FAPi is quantitatively prepared in an instant kit-type version at room temperature. It demonstrated high stability in human serum, affinity for FAP in the low nanomolar range, and high internalization rate when associated with CAFs. Biodistribution and PET studies in prostate and glioblastoma xenografts revealed high and specific tumor uptake. Elimination of the radiotracer mainly occurred through the urinary tract. The clinical data are in accordance with the preclinical data concerning the organ receiving the highest absorbed dose (urinary bladder wall, heart wall, spleen, and kidneys). Different to the small-animal data, uptake of [^68^Ga]Ga-DATA^5m^.SA.FAPi in tumor lesions is rapid and stable and tumor-to-organ and tumor-to-blood uptake ratios are high.

**Conclusion:**

The radiochemical, preclinical, and clinical data obtained in this study strongly support further development of [^68^Ga]Ga-DATA^5m^.SA.FAPi as a diagnostic tool for FAP imaging.

**Supplementary Information:**

The online version contains supplementary material available at 10.1007/s00259-023-06285-2.

## Introduction

Most tumors develop as solid tissue masses with a distinct structure, encompassed by two strongly associated compartments: neoplastic cells and stroma. Stroma is induced by the neoplastic cells which are further dispersed in it. For the tumors to grow above a minimum size of 1 to 2 mm, the creation of stroma is necessary since it mainly includes connective tissue, the basement membrane, fibroblasts, extracellular matrix, immune cells, and blood vessels. Even though stromal components hold certain tumor-suppressing abilities, stroma appears to change during the malignancy. Eventually the interactions between the cancer and stromal cells promote growth, invasion, and metastasis. The increased number of fibroblasts contained in stroma are pathologically activated within the course of the disease, forming the so-called cancer associated fibroblasts (CAFs) [1]. CAF activation may be influenced by several stimuli, both cellular and environmental, leading to a variety of CAF phenotypes [2], that are identified by several biomarkers expressed on their surface [3]. CAFs appear to have an abundant expression of fibroblast activation protein (FAP), also known as prolyl endopeptidase FAP or seprase, associated with fibrosis, tissue repair, inflammation, and extracellular matrix (ECM) degradation. FAP is also involved in tumor growth, invasion, metastasis and immunosuppression [4].

FAP is overexpressed in malignant tissue on activated fibroblasts on a variety of malignant tumors such as breast, colorectal, pancreatic, melanoma, myeloma, gastric, brain, and ovarian carcinomas [5–11] while it shows less abundance in normal healthy tissue. Hence, FAP radioligands are considered important tools for in vivo tumor imaging and/or therapy [12].

**Fig. 1:**
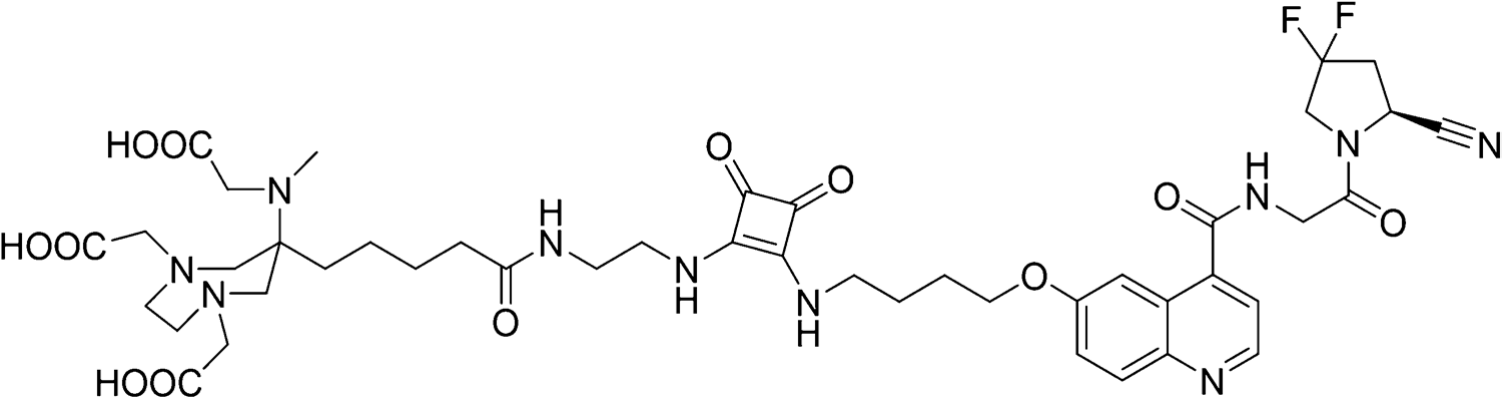
Chemical structure of DATA^5m^.SA.FAPi

With the view to develop pan-cancer theranostic radiotracers, several research groups have shifted their scientific focus on imaging and/or treating various tumors by targeting not directly the cancer cells but FAP^+^ CAFs [1]. Many efforts have been made in the last years, and several FAP-targeting radiotracers, derived from antibodies, FAP-inhibitors (FAPi) and peptides, have shown great promise in preclinical [13–26] and clinical settings [13–15, 20, 21, 23, 27–35]. The FAP inhibitor UAMC1110, due to its high potency and selectivity towards FAP, appears to be a highly suitable candidate for the development of FAP-based radiopharmaceuticals [36]. For radiolabeling with radiometals, UAMC1110 is functionalized with a matching chelator. Although, in principle, many different chelators are available for gallium-68, the chelator DATA^5m^ is particularly attractive since it can be conveniently labeled at room temperature (RT) [37]. Coupling of DATA^5m^ to UAMC1110 via a squaric acid based spacer has resulted in the lead candidate DATA^5m^.SA.FAPi (Fig. 1) [18, 19, 38].

This study aimed at determining a set of preclinical characteristics of [^68^Ga]Ga-DATA^5m^.SA.FAPi, which appeared to be relevant in the course of the ongoing clinical applications [39–41] of this tracer:

We evaluated [^68^Ga]Ga-DATA^5m^.SA.FAPi in vitro including stability studies, metabolite analysis, protein binding, and internalization studies. [^68^Ga]Ga-DATA^5m^.SA.FAPi was further evaluated in vivo and ex vivo in glioblastoma and prostate xenografts by biodistribution and PET/CT imaging studies. Further on, the PET/CT data from six patients suffering from metastasized castration resistant prostate cancer (mCRPC) were analyzed to compare small-animal and patient uptake profiles. In preparation of therapeutical applications, biodistribution, and biokinetics in the organs potentially at risk when using [^68^Ga]Ga-DATA^5m^.SA.FAPi were determined including dosimetry.

## Material and methods

The supplier information for all reagents and details of instruments used are provided in the supplemental data.

### Radiochemistry/quality control of the radiotracer/stability

[^68^Ga]Ga-DATA^5m^.SA.FAPi was prepared as described in the supplemental data. Chemical, radiochemical purity and stability for a period of 4 h post labelling was determined by Reversed Phase High Performance Liquid Chromatography (RP-HPLC).

### Lipophilicity/protein binding studies and metabolic stability in human serum

The lipophilicity, protein binding, and metabolic stability of [^68^Ga]Ga-DATA^5m^.SA.FAPi in human serum were determined (supplemental data).

### Cell lines/Western blot analysis

The cultivation of the human prostate adenocarcinoma cell line PC3, the human glioblastoma cell line U87MG, the cancer associated prostate fibroblast cell line CAF, and the western blot experiments are described in the supplemental data.

### Radioligand binding assays

For radioligand binding assays, cells were incubated with approximately 2.5 pmol of [^68^Ga]Ga-DATA^5m^.SA.FAPi for 120 min at 4 °C (supplemental data).

### Saturation binding studies/internalization studies

For saturation studies, cells were incubated with increasing concentrations of ^68/nat^Ga-DATA^5m^.SA.FAPi (0.1–10 nM). For internalization studies, approximately 2.5 pmol of [^68^Ga]Ga-DATA^5m^.SA.FAPi were added to the cells followed by incubation for 15, 30, 60, 90, 120, 180 and 240 min at 37 °C, 5% CO_2_ (supplemental data).

### Animal models

#### U87MG and PC3 xenografts

Female athymic Balb/C nude mice (age: 6 weeks, weight: 16–20 g) and male athymic Balb/C nude mice (age: 6 weeks, weight: 20–25 g) were implanted with U87MG and PC3 cells (5 × 10^6^ in 100 µL PBS) into their right shoulder, respectively. After an average of 10–12 and 15–17 days, tumor size reached 150 to 200 mg and 250 to 300 mg for U87MG and PC3 xenografts, respectively and the animals were used for biodistribution and PET/CT imaging studies.

### Biodistribution/small-animal PET/CT imaging

Ten pmol (0.06–0.09 MBq) of [^68^Ga]Ga-DATA^5m^.SA.FAPi in 100 µL of NaCl 0.9% were injected intravenously into the tail vein of U87MG or PC3 xenografts. Animals were terminally anesthetized by intraperitoneal injection of an overdose of pentobarbital sodium (150 mg/kg) followed by biodistribution studies. PET images were obtained upon injection of 200 pmol of [^68^Ga]Ga-DATA^5m^.SA.FAPi (1.2–1.5 MBq/100 μL) on U87MG or PC3 xenografts (supplemental data).

### Clinical assessment of [^68^Ga]Ga-DATA^5m^.SA.FAPi in prostate cancer patients

As part of the evaluation of potential therapy with a therapeutic FAPi ligand in patients with mCRPC without other treatment options, sequential imaging with [^68^Ga]Ga-DATA5m.SA.FAPi was performed in 6 patients over a period of up to 3.5 h to determine the target expression and the stability of the radioligand. We also used the data acquired in this process to perform incorporation dosimetry for the PET tracer. A detailed description with regard to the study design, selection of patients, PET/CT imaging, dosimetry/calculation of the absorbed doses is provided in the supplemental data.

### Statistical analysis

All the data are expressed as the mean of values ± standard deviation (mean ± SD). Prism 8 Software (GraphPad Software) was used to determine statistical significance at the 95% confidence level, with a *P* value of less than 0.05, considered significant.

## Results

### Chemistry and radiochemistry/quality control of the radiotracer/stability

DATA^5m^.SA.FAPi (Fig. 1) was synthesized as described previously [18]. The corresponding analytical data are presented in Table 1.

**Table 1:** Analytical data of DATA^5m^.SA.FAPi

Compound	Elemental composition	Purity^§^	Calculated molecular monoisotopic mass	MS (ESI,negative mode)	Rt (min)
DATA^5m^.SA.FAPi	C_44_H_56_F_2_N_10_O_12_	> 95%	954.4	953.4 m/z	13.4

^§^Based on RP-HPLC, UV detection at 214 nm, (supplemental data)

DATA^5m^.SA.FAPi was successfully labelled in less than 5 min with gallium-68 at RT in  > 98% radiochemical purity as determined by RP-HPLC (Fig. 1Sa, supplemental data). The apparent molar activity (A_m_) was ranging between 15 and 22 GBq/µmol. The isolated radiochemical yield was 80 ± 3% not decay corrected (*n* = 10). No formation of colloids was observed. Radiotracer stability over time was also determined and neither radiolysis nor decomposition was observed for a period of 4 hpost labeling.

### Lipophilicity/protein binding studies and metabolic stability in human serum

With a LogD_Octanol/PBS_ of -3.6 ± 0.1, the radiotracer showed a hydrophilic profile. To estimate the bioavailability of [^68^Ga]Ga-DATA^5m^.SA.FAPi in circulation, the extent of human serum protein binding was determined. After 30 min, 9.9 ± 0.5% of the incubating gallium-68 activity was bound to serum proteins.

The metabolic stability of [^68^Ga]Ga-DATA^5m^.SA.FAPi in human serum was monitored by RP-HPLC. Apart from minute amounts of a polar metabolite (< 0.5%) no change in the chromatogram pattern was observed after 30 min of incubation at 37 °C (Fig. 1Sb, supplemental data).

### Western blot analysis

**Fig. 2:**
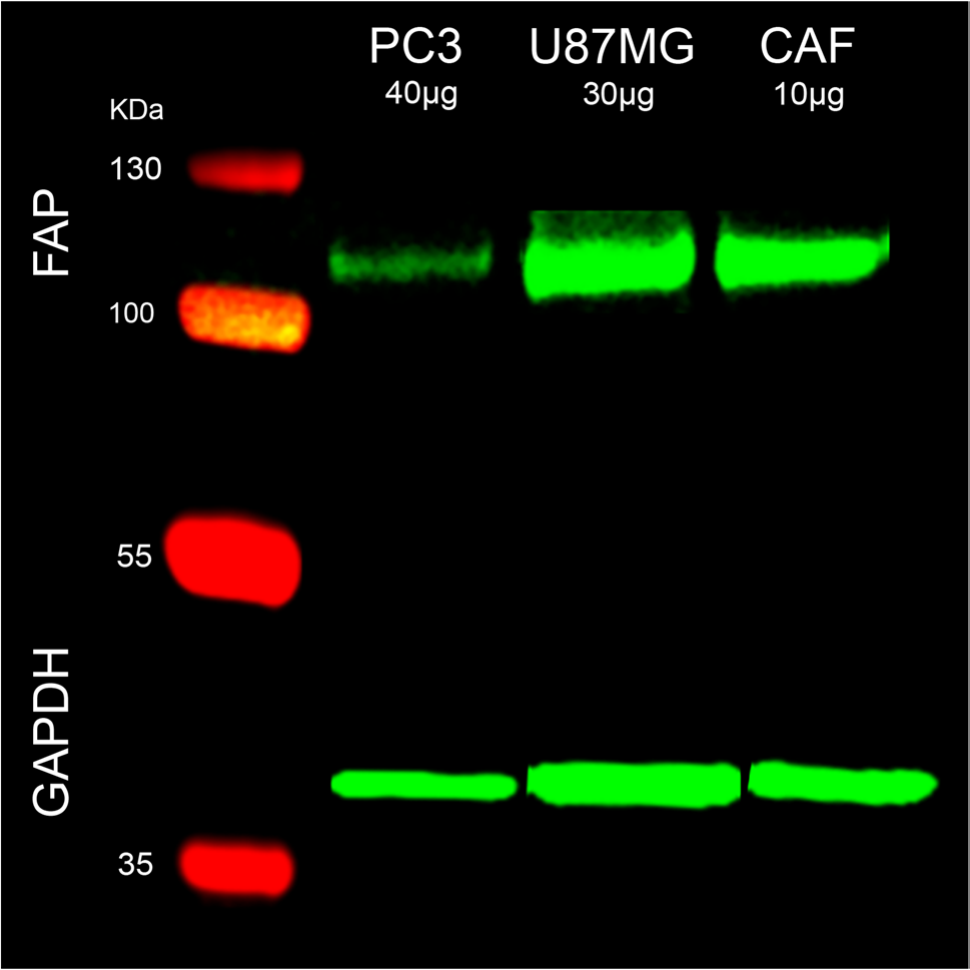
Extracts from PC3, U87MG and CAF were separated and incubated with monoclonal anti-human FAP and GAPDH antibodies followed by staining with Anti IgG H&L, and IRDye®

Whole-cell extracts from PC3, U87MG, and CAF cells were subjected to Western blot to identify their FAP expression (Fig. 2). The FAP protein level varies between the three tested cell lines. CAF had a higher FAP protein level followed by U87MG and PC3.

### Radioligand binding assay

Radioligand binding assays were performed to verify the FAP expression on the surface of PC3, U87MG and CAF cells. The percentage of the specific cell surface associated activity was 0.18 ± 0.07, 3.4 ± 0.46 m and 23.69 ± 0.65, respectively.

### Saturation/internalization studies

**Fig. 3:**
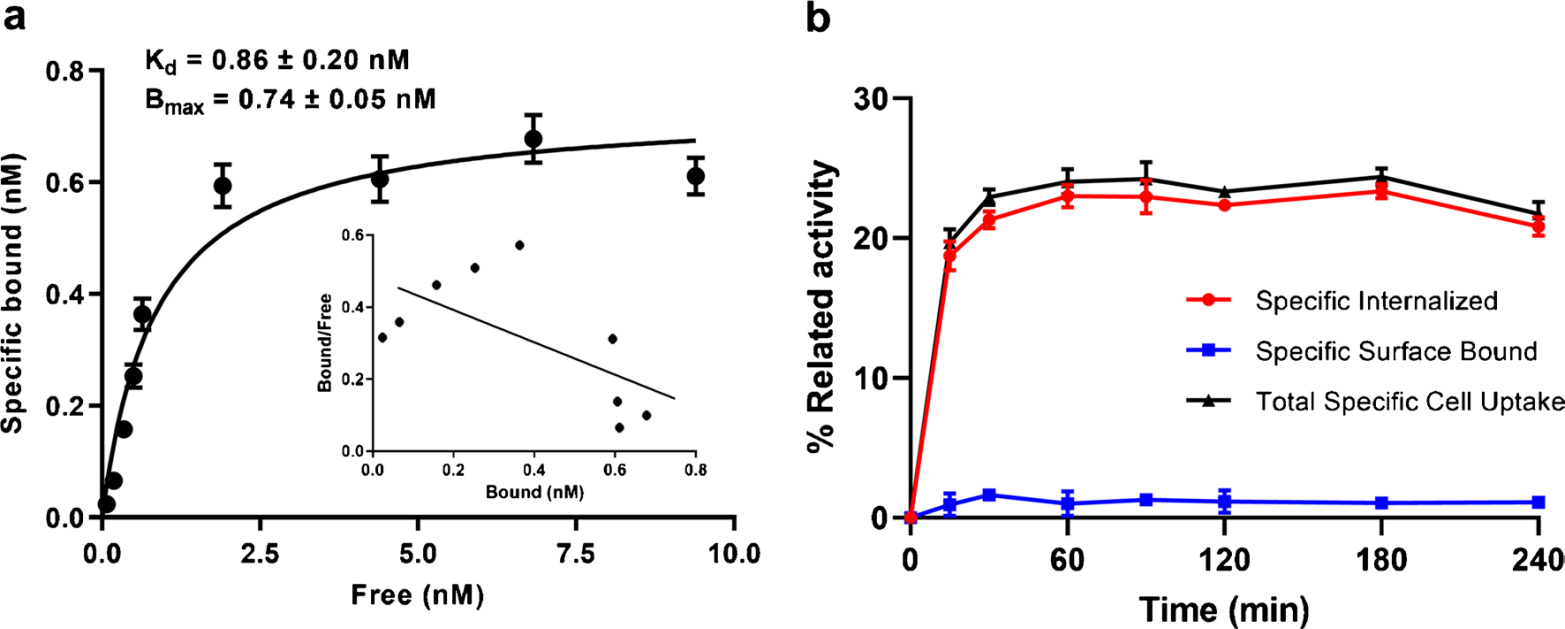
**a** Saturation binding study on CAF cells, using increasing concentrations of ^68/nat^Ga-DATA^5m^.SA.FAPi (0.1 to 10 nM). Dissociation constant (K_d_) and maximum number of binding sites (B_max_) were calculated from nonlinear regression analysis using GraphPad Prism, **b** Internalization rate and specific surface bound uptake after the incubation of CAFs with [^68^Ga]Ga-DATA^5 m^.SA.FAPi within 4 h at 37 °C. Total specific cell uptake calculated as specific surface bound fraction plus specific internalized fraction. Total specific cell uptake is expressed as percentage of the total applied radioactivity. Nonspecific binding was determined in the presence of 1 μM UAMC1110

^68/nat^Ga-DATA^5m^.SA.FAPi was well associated with the CAFs within the incubation period (Fig. 3b). Exposure of the cells to the radiotracer resulted in a steep increase of total cell associated uptake (> 15% after 15 min), which gradually increased during further incubation up to 240 min. The highest total specific cell uptake of ^68/nat^Ga-DATA^5m^.SA.FAPi was 24.4 ± 0.6%. Fast and high internalization rate was also observed. Already after 15 min of incubation, approximately 95% of the total cell associated activity was internalized in the cells. Blocking experiments performed with excess of UAMC1110, showed negligible nonspecific binding on the cell surface demonstrating the high specificity ^68/nat^Ga-DATA^5m^.SA.FAPi towards CAFs.

^68/nat^Ga-DATA^5m^.SA.FAPi exhibited high affinity for FAP^+^ CAFs, with a K_d_ value of 0.86 ± 0.20 nM (Fig. 3a). The B_max_ was 0.74 ± 0.05 nM which correspond to approximately 450000 receptors per cell.

### Biodistribution studies

Ex vivo biodistribution data and tumor-to-tissue ratios of [^68^Ga]Ga-DATA^5m^.SA.FAPi are summarized in Table 2. At 1 h after injection, the overall pharmacokinetic performance of the radiotracer in both tumor models follows the same trend, but blood and muscle values for PC3 are significantly lower (*P* values of 0.005 and 0.0106, respectively). High blood accumulation was observed 1 h after injection in both cases, (11.3 ± 0.9 and 8.2 ± 0.8% IA/g for U87MG and PC3 xenografts, respectively) which was gradually reduced after 3 h (5.0 ± 0.7 and 3.9 ± 0.6% IA/g for U87MG and PC3 xenografts, respectively). Tumor uptake is specific with values of approximately 6.6 and 5% IA/g at 1 h p.i. for U87MG and PC3 xenografts respectively, and only slow washout 3 h after injection. Elevated uptake of [^68^Ga]Ga-DATA^5m^.SA.FAPi was found in/around the bones. The radiotracer is mainly eliminated through the urinary tract for both tested xenografts. Receptor binding specificity was demonstrated by a more than 85% reduction of uptake in the tumor and receptor positive organs after co-injection of 2000-fold excess of UAMC1110.

**Table 2:** Biodistribution data of [^68^Ga]Ga-DATA^5m^.SA.FAPi in U87MG and PC3 xenografts, 1 h, 2 h, and 3 h along with blocking experiments 2 h after injection

	U87MG				PC3			
Organ	1 h	2 h	3 h	2-h blocking	1 h	2 h	3 h	2-h blocking
Blood	11.3 ± 0.9	6.6 ± 0.5	5.0 ± 0.7	0.4 ± 0.1	8.2 ± 0.8	6.2 ± 0.9	3.9 ± 0.6	0.8 ± 0.0
Heart	5.6 ± 0.8	3.7 ± 0.6	3.5 ± 1.4	0.4 ± 0.0	3.1 ± 0.4	2.6 ± 0.1	2.2 ± 0.3	0.5 ± 0.1
Liver	3.6 ± 0.4	3.5 ± 0.2	3.1 ± 1.0	0.3 ± 0.0	2.7 ± 0.4	2.5 ± 0.3	2.4 ± 0.1	0.4 ± 0.0
Spleen	2.8 ± 0.7	1.9 ± 0.3	1.8 ± 0.2	0.3 ± 0.1	2.1 ± 0.3	1.6 ± 0.1	2.1 ± 0.7	0.6 ± 0.1
Lung	4.6 ± 0.9	3.3 ± 0.3	2.5 ± 0.2	0.9 ± 0.2	3.6 ± 0.7	2.7 ± 0.5	2.5 ± 0.5	0.6 ± 0.1
Kidney	4.6 ± 0.4	3.6 ± 0.7	3.7 ± 1.1	1.3 ± 0.2	3.2 ± 0.4	2.5 ± 0.4	2.5 ± 0.4	0.9 ± 0.1
Stomach	2.2 ± 0.8	1.8 ± 0.4	1.8 ± 0.5	0.4 ± 0.1	2.0 ± 0.4	1.6 ± 0.3	2.0 ± 0.3	0.4 ± 0.1
Intestine	5.1 ± 1.0	4.6 ± 1.8	4.9 ± 0.6	1.2 ± 0.7	4.2 ± 0.8	3.2 ± 0.8	2.8 ± 0.1	0.9 ± 0.0
Pancreas	9.9 ± 3.2	7.1 ± 1.3	6.9 ± 1.0	0.5 ± 0.1	7.4 ± 1.9	5.3 ± 0.3	3.7 ± 0.6	0.3 ± 0.1
Muscle	4.2 ± 0.9	2.8 ± 0.8	3.5 ± 0.9	1.3 ± 1.4	2.0 ± 0.3	2.0 ± 0.2	2.2 ± 0.2	0.4 ± 0.1
Bone	4.5 ± 1.4	4.7 ± 1.2	6.1 ± 1.2	2.0 ± 0.3	3.2 ± 0.9	2.5 ± 0.4	5.7 ± 0.3	1.5 ± 0.9
Tumor	6.6 ± 1.6	6.0 ± 1.0	5.4 ± 1.0	1.1 ± 0.4	5.0 ± 0.6	4.6 ± 0.8	4.7 ± 0.3	0.7 ± 0.0
Tumor/blood	0.6 ± 0.2	0.9 ± 0.2	1.1 ± 0.4		0.6 ± 0.1	0.7 ± 0.1	1.2 ± 0.2	
Tumor/liver	1.8 ± 0.5	1.7 ± 0.2	1.7 ± 0.2		1.9 ± 0.4	1.8 ± 0.1	1.9 ± 0.2	
Tumor/kidney	1.4 ± 0.4	1.7 ± 0.4	1.5 ± 0.3		1.6 ± 0.3	1.8 ± 0.1	1.9 ± 0.1	
Tumor/muscle	1.6 ± 0.5	2.1 ± 0.7	1.5 ± 0.3		2.5 ± 0.1	2.2 ± 0.2	2.1 ± 0.1	
Tumor/pancreas	0.7 ± 0.2	0.9 ± 0.3	0.8 ± 0.4		0.7 ± 0.3	0.9 ± 0.1	1.3 ± 0.3	

Data are expressed in percentage of injected activity per gram of tissue (% IA/g) and are presented as the mean ± SD (*n* = 4)

### Small-animal PET/CT studies

**Fig. 4:**
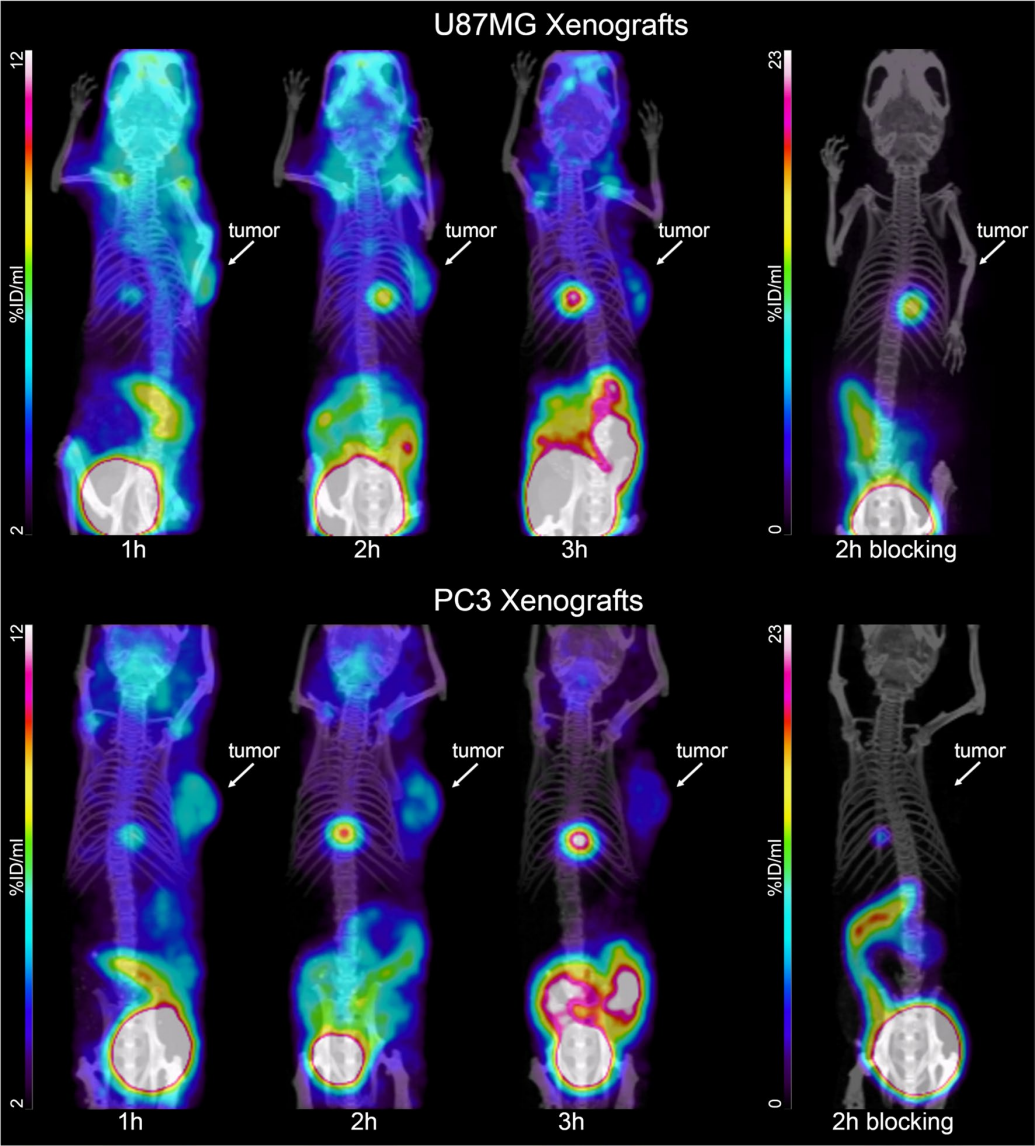
PET/CT images of U87MG and PC3 xenografts upon injection of [^68^ Ga]Ga-DATA^5m^.SA.FAPi at 1, 2 and 3 h along with blocking studies at 2 h after injection

Representative PET/CT images were acquired 1, 2, and 3 h after injection of [^68^Ga]Ga-DATA^5m^.SA.FAPi in PC3 and U87MG xenografts (Fig. 4). The radiotracer is specifically taken up by the FAP-positive organs. The tumors are well visualized and the uptake in the other organs matches the results of the ex vivo biodistribution studies. Specific tumor uptake was verified by blocking experiments. Notably, also the background, which is mainly dominated by high blood pool and joints uptake, is blockable.

**Fig. 5:**
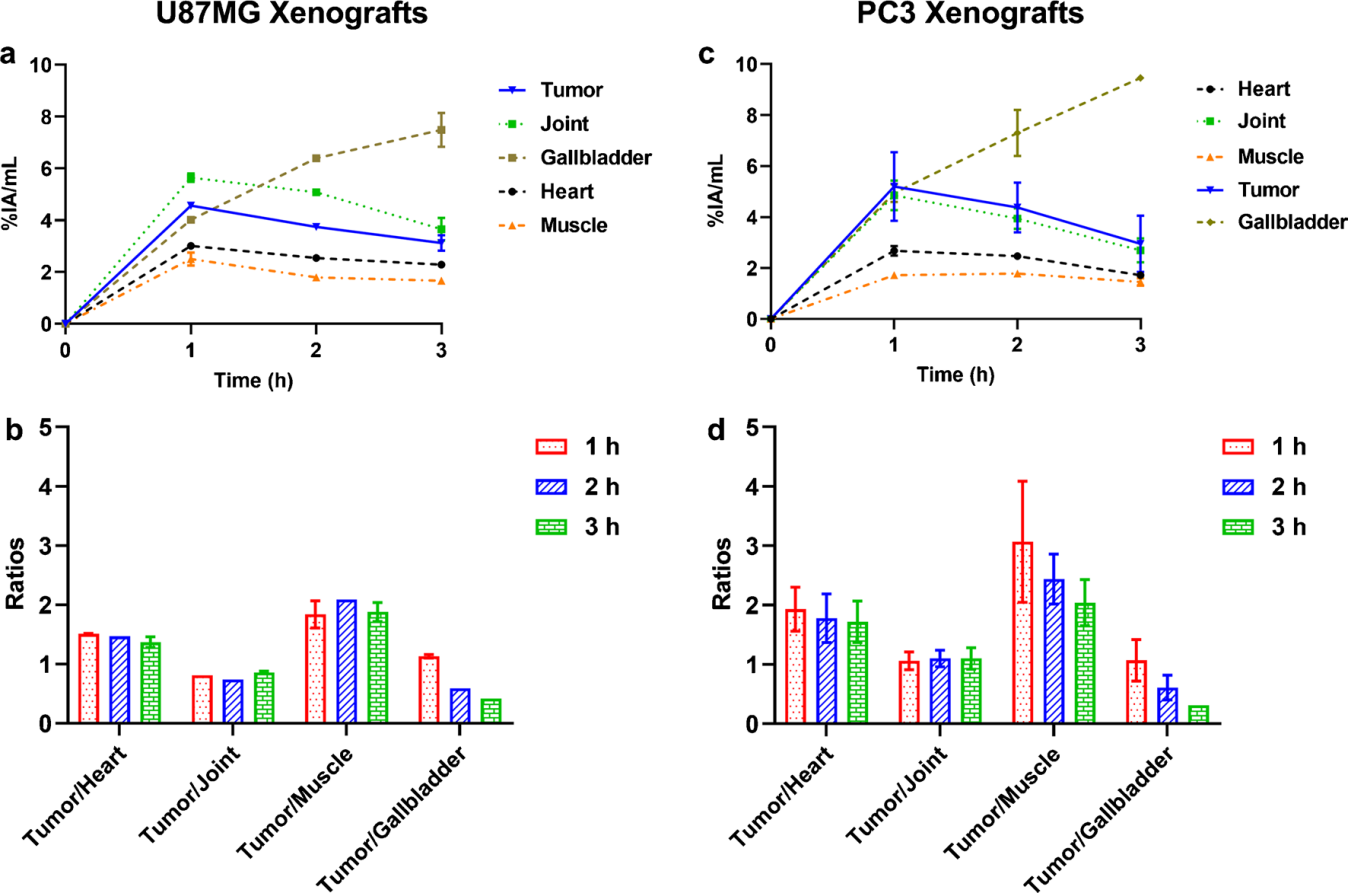
Quantitative analysis of the PET images (**a**, **b**) and tumor-to-background ratios (**c**, **d**) for U87MG and PC3 xenografts, respectively

A quantitative analysis of the PET/CT images including pharmacokinetics (tissue uptake and tumor-to-tissue ratios) is presented in Fig. 5. In general, the tumor-to-tissue ratios for PC3 xenografts are higher than those found for U87MG xenografts (Fig. 5).

### Clinical assessment of [^68^Ga]Ga-DATA^5m^.SA.FAPi in prostate cancer patients

**Fig. 6:**
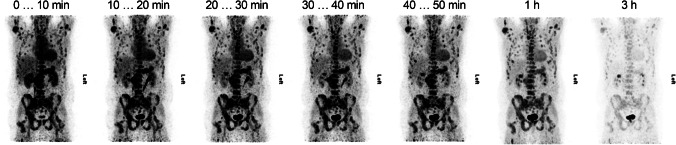
Maximum-intensity-projections of the PET images of a patient (Pat. 6) from the base of the skull to the mid-thigh including whole-body CT imaging. The first 6 PETs were taken within the first 1 h, starting with the injection and covering a time span of 10 min each. Prior to this series the corresponding auxiliary CT was acquired. The seventh PET was started after 3 h, with an acquisition time of 2.5 min per bed. For this series, a separate auxiliary CT was acquired

**Fig. 7:**
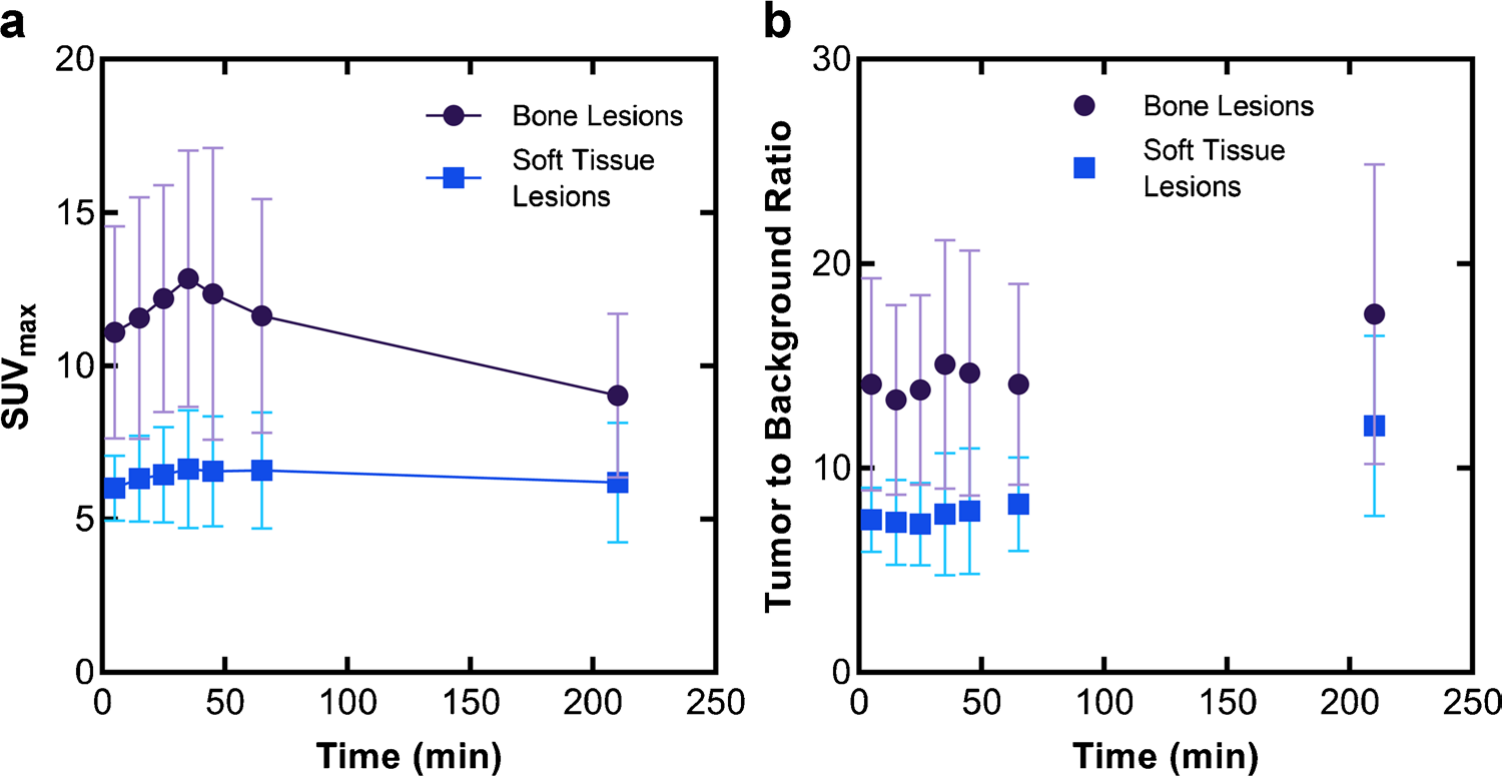
Uptake (SUV_max_) of [^68^Ga]Ga-DATA^5m^.SA.FAPi in bone and soft tissue tumor lesions (**a**) and a constant tumor-to-background-ratio (**b**) indicating stable binding of the radioligand

As in the preclinical studies, patient use showed rapid and specific uptake of the tracer and, as with most ^68^Ga-based tracers, it is predominantly renally excreted. An example of the temporal distribution of [^68^Ga]Ga-DATA^5m^.SA.FAPi in a patient is shown in Fig. 6. An overview of the maximum-intensity-projections (MIP) for all patients and all time points is included in the supplemental data (Fig. 3S). In terms of uptake in tumor lesions, [^68^Ga]Ga-DATA5m.SA.FAPi shows a stable tumor to background ratio in addition to rapid uptake, with higher uptake in osseous lesions (Fig. 7).

**Fig. 8:**
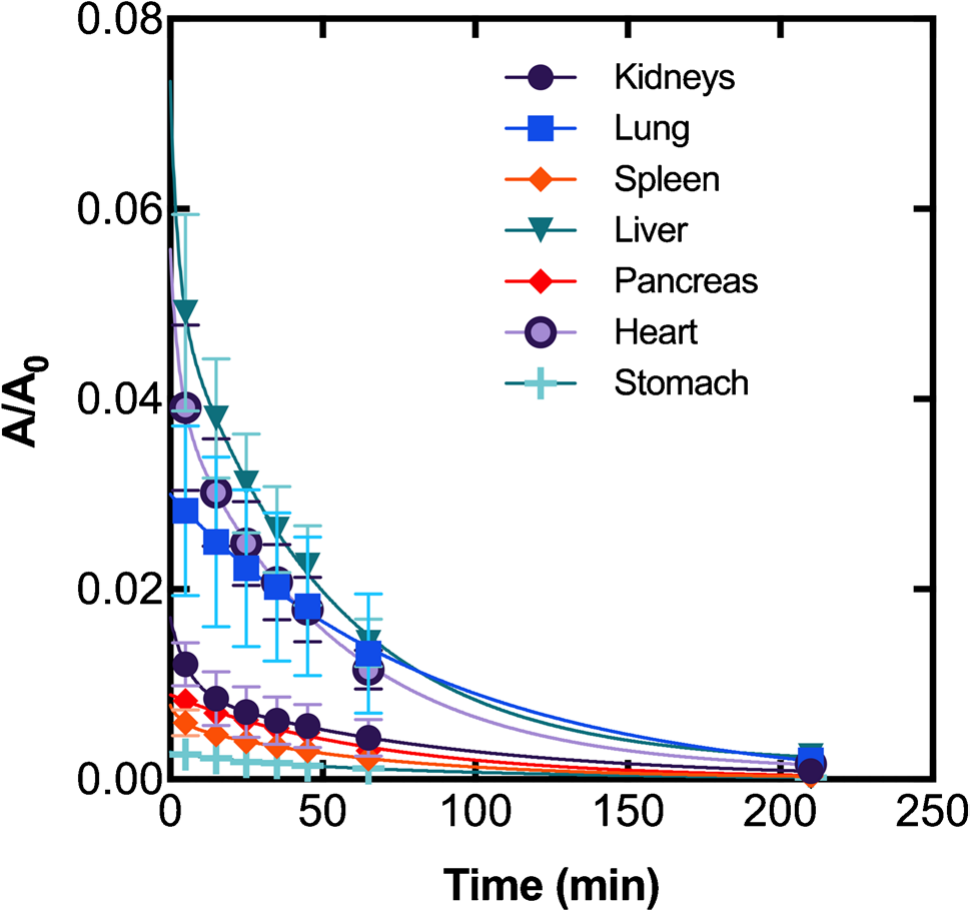
Relative Uptake (time-activity curves) of [^68^Ga]Ga-DATA^5m^.SA.FAPi for relevant source organs with normal physiological uptake

Time-activity curves for relevant source organs are shown in Fig. 8; a more detailed graphic depicting the time-activity curve for each source organ is part of the supplemental data (Fig. 4S). Table 3 summarizes the absorbed doses of all organs and the effective dose averaged over all 6 patients. The organ receiving the highest absorbed dose was the urinary bladder wall (67 μGy/MBq), followed by the heart wall, the spleen, and the kidneys (26.3, 17.5, and 13.9 μGy/MBq respectively). A detailed overview for each patient and the underlying Time-Integrated-Activity-Coefficients (TIAC) are summarized in the supplemental data (Tables 2S and 3S).

**Table 3:** Mean absorbed doses of all organs and the effective dose (ED) averaged over all 6 patients

Target organ	Beta (mGy/MBq)	Gamma (mGy/MBq)	Total (mGy/MBq)	ICRP-103 ED (mSv/MBq)
Adrenals	7.69E − 03	5.59E − 03	1.33E − 02	1.23E − 04
Brain	5.83E − 03	2.42E − 03	8.24E − 03	8.24E − 05
Esophagus	5.83E − 03	4.70E − 03	1.05E − 02	4.21E − 04
Eyes	5.83E − 03	2.42E − 03	8.25E − 03	0.00E + 00
Gallbladder wall	6.17E − 03	4.88E − 03	1.10E − 02	1.02E − 04
Left colon	5.90E − 03	4.54E − 03	1.05E − 02	5.07E − 04
Small intestine	5.86E − 03	4.51E − 03	1.04E − 02	9.58E − 05
Stomach wall	8.88E − 03	4.97E − 03	1.39E − 02	1.66E − 03
Right colon	5.83E − 03	4.32E − 03	1.02E − 02	4.93E − 04
Rectum	5.83E − 03	4.79E − 03	1.06E − 02	2.44E − 04
Heart wall	1.96E − 02	6.72E − 03	2.63E − 02	2.43E − 04
Kidneys	1.14E − 02	2.57E − 03	1.39E − 02	1.29E − 04
Liver	9.50E − 03	4.95E − 03	1.45E − 02	5.78E − 04
Lungs	2.49E − 02	4.86E − 03	2.98E − 02	3.57E − 03
Pancreas	3.38E − 02	8.02E − 03	4.18E − 02	3.86E − 04
Prostate	5.83E − 03	5.23E − 03	1.11E − 02	5.11E − 05
Salivary glands	5.83E − 03	3.11E − 03	8.94E − 03	8.94E − 05
Red marrow	4.15E − 03	3.83E − 03	7.98E − 03	9.57E − 04
Osteogenic cells	3.50E − 03	3.74E − 03	7.24E − 03	7.24E − 05
Spleen	1.29E − 02	4.56E − 03	1.75E − 02	1.62E − 04
Testes	5.83E − 03	3.14E − 03	8.97E − 03	3.59E − 04
Thymus	5.83E − 03	4.67E − 03	1.05E − 02	9.69E − 05
Thyroid	5.83E − 03	3.78E − 03	9.60E − 03	3.84E − 04
Urinary bladder Wall	5.63E − 02	1.07E − 02	6.70E − 02	2.68E − 03
Total body	7.23E − 03	3.11E − 03	1.03E − 02	0.00E + 00
Effective dose ED				1.35E − 02
(ICRP 103)				

## Discussion

[^68^Ga]Ga-DATA^5m^.SA.FAPi, previously reported by Moon et al., provided affinity and selectivity data towards FAP [18, 19]. Herewith, our goal was to evaluate the performance of [^68^Ga]Ga-DATA^5m^.SA.FAPi by investigating, preclinically and clinically, its ability to detect FAP-positive tumors of different origin.

The coupling of DATA^5m^ via the squaric acid-based spacer to UAMC1110 and the radiolabeling with gallium-68 did not influence UAMC1110 high FAP binding affinity as well as its in vivo stability. In our saturation binding assay, ^68/nat^Ga-DATA^5m^.SA.FAPi showed extremely high affinity towards FAP on CAFs, with a K_d_ value in the sub-nanomolar range. Additionally, more than 95% of the radioactivity in human serum corresponds to intact radiotracer indicating that the radiotracer is not subjected to in vivo metabolic degradation. The enhanced affinity towards FAP may have also resulted in the fast internalization rate.

[^68^Ga]Ga-DATA^5m^.SA.FAPi, most probably due to its balanced lipophilicity, delivers biodistribution and PET images which advocate specific uptake by both experimental tumor. Elimination took place mainly via the urinary tract with a lower residence in the kidneys and higher in the urinary bladder wall both in mice and humans. UAMC1110 small size might be responsible for the low kidney uptake, which seems to be a superiority of the UAMC1110-based radiotracers compared to the peptidomimetic-based FAP targeting counterparts where it was shown that kidney was the organ with the highest non-target uptake at early time points [24]. The gradually increasing uptake in the gallbladder, as indicated by PET images, may also suggest a second excretion pathway. Another point, which is in accordance with the findings for other radiolabeled FAP inhibitors concerns the non-specific accumulation of [^68^Ga]Ga-DATA^5m^.SA.FAPi in non-target organs including muscles, lung, pancreas and joints [13–15, 17, 20, 21]. This context suggests that fibroblasts involved in the development of those organs may also be detected by FAP targeting. Additionally, the relatively high blood pool uptake by the radiolabeled FAP- inhibitors in general is one of the challenges. Studies have shown the presence of a soluble FAP form in both bovine [42] and human plasma [43]. Up to date, it is not clear which is the function of this soluble form; if, for example, there is any interaction with the full-length membrane-bound FAP form or whether the presence of the soluble FAP form is the result of shedding from the membrane surface or the biosynthesis of alternative splicing. The understanding of how FAP enters the circulation but also how and to which extent, FAP targeting might be influenced when using nuclear probes, is of paramount importance to strengthen the development of FAP-based radiotracers and improve the quality of the current nuclear imaging applications.

The PC3 and U87MG subcutaneous xenografts were used because of their modest tumor growth and the abundant FAP expression in the stroma of human prostate carcinomas [27, 28] and glioblastomas [22, 44]. However, in cancer cell-derived xenografts, tumor growth requires the development of tumor microenvironment and the recruitment of murine fibroblasts. Due to the similar functional homology and the 89% shared sequence identity between murine and human-FAP, studies have shown that murine-FAP also promotes tumor growth in cell derived xenografts [45]. Our in vitro data showed low FAP protein expression for U87MG and even lower for PC3 cells compared to CAF. The radioligand binding assays confirmed high FAP cell-surface expression for CAF, very low for U87MG and negligible for PC3. However, under in vivo conditions, where neither extended vascularization nor blood pool activity in the tumors was observed, the radiotracer is specifically taken up by both experimental tumors. Further studies are required to verify if FAP cell-surface expression is limited to the murine stroma, or if the initially FAP-surface low expressing (U87MG) or negative tumor cells (PC3) turned out to be FAP-positive when exposed to murine CAFs. The murine origin of tumor stroma in xenotransplanted mice is certainly a limitation since the tumor environment and molecular signature of the corresponding human tumor are not retained. Nevertheless, the successful FAP targeting by [^68^Ga]Ga-DATA^5m^.SA.FAPi provides a strong evidence of the otherwise successful strategy of tumor stroma targeting.

Patient-derived xenografts (PDXs) might represent a more suitable model for FAP targeting in preclinical settings, however, special attention should be paid, since only the PDXs established by direct implantation of fresh surgical tissue fragments into mice mimic the human conditions. After a couple of passages murine fibroblasts are recruited and tumor stroma is eventually dominated by them.

The encouraging in vitro and in vivo preclinical data of [^68^Ga]Ga-DATA^5m^.SA.FAPi was the driving force to immediately plan and execute a study aiming on acquiring initial clinical experience on human prostate cancer patients and verifying if the preclinical data are translated to humans. These data were also collected as part of the preparation for potential FAPi-based therapy. [^68^Ga]Ga-DATA^5m^.SA.FAPi is mainly eliminated via the urinary tract. Considerable uptake in organs presenting FAP expression and tumor lesions was observed, which makes the images easy to interpret. The optimum time for PET/CT appears to be 40–50 min after the administration of the tracer. Interestingly, the absolute tumor uptake values in animals (% activity/gram; Table 2, Fig. 5) are slightly lower in patients (SUV_max_; Fig. 7). More importantly, the tumor-to-organ and tumor-to-background ratios, respectively, are much higher in patient studies compared to the preclinical data. These non-mirroring findings may be due to the mixture of both species, human- and murine-FAP on the experimental tumors. The fact that the small animal PET modalities have not exactly the same technical specifications with the clinical scanners might be a second reason for the non-direct translation of preclinical imaging findings to clinical systems.

The clinical biodistribution and kinetics data published for [^68^Ga]Ga-DATA^5m^.SA.FAPi [39–41]are comparable to other FAPi tracers [29, 32, 46]. As with these, the high tumor-to-background ratio achieved after only 10 min enables early imaging with [^68^Ga]Ga-DATA^5m^.SA.FAPi, with the contrast ratio still improving over time. This opens a wide time window for imaging, which can thus be adapted to clinical needs. The average effective dose for the administration of 200 MBq of [^68^Ga]Ga-DATA^5m^.SA.FAPi was 2.7 mSv, which is slightly higher compared to the reported values for other FAPi tracers (e.g. 1.56 mSv and 2.2 mSv for [^68^Ga]Ga-FAPI-46 [46] and [^68^Ga]Ga-DOTA.SA.FAPi [32], respectively), but without being associated with a higher risk in use. In principle, the results of incorporation dosimetry are comparable to those of other ^68^Ga-labelled tracers, e.g. tracers targeting prostate-specific membrane antigens or somatostatin receptors [47–49].

In conclusion, the preclinical and clinical data presented in this work, in particular also the data related to calculation of the incorporation dosimetry for [^68^Ga]Ga-DATA^5m^.SA.FAPi, will support further clinical application of this DATA^5m^.SA-functionalized FAP inhibitor with superior radiolabeling properties. One main advantage of DATA^5m^.SA.FAPi compared to other reported FAPi-based precursors is due to its functionalization with the chelator DATA^5m^ which allows the preparation of the ^68^Ga-labelled radiotracer in an “advanced” kit-type protocol since fast and efficient radiolabeling can be achieved at ambient temperature [37].

### Supplementary Information

Below is the link to the electronic supplementary material.

Supplementary file1 (DOCX 2.13 MB)

## Data Availability

All data generated or analyzed during this study are included in this published article and its supplementary information files.
