# Supplementary material for: Translational assessment of a DATA-functionalized FAP inhibitor with facile 68Ga-labeling at room temperature
Source: Eur J Nucl Med Mol Imaging. 2023 Jun 7;50(11):3202–13. doi: 10.1007/s00259-023-06285-2 (PMC10541845; doi:10.1007/s00259-023-06285-2)
Supplement: Supplementary file 1 — Supplementary file1 (DOCX 2.13 MB) [file 259_2023_6285_MOESM1_ESM.docx]

**Supplemental Data**

**Translational assessment of a DATA functionalized FAP inhibitor with facile ^68^Ga-labeling at room temperature**

Alondra Escudero-Castellanos^1*^, Jens Kurth^2*^, Surachet Imlimthan^1^, Elena Menéndez^1^, Eirinaios Pilatis^1^, Euy Sung Moon^3^, Tilman Läppchen^1^, Hendrik Rathke^1^, Sarah M Schwarzenböck^2^, [Bernd J Krause](https://pubmed.ncbi.nlm.nih.gov/?term=Krause+BJ&cauthor_id=34359784)^2^, Frank Rösch^3^, Axel Rominger^1^, Eleni Gourni^1^

*^1^Department of Nuclear Medicine, Inselspital, Bern University Hospital, University of Bern, Bern, Switzerland*

*^2^Department of Nuclear Medicine, Rostock University Medical Centre, Rostock, Germany*

*^3^Department of Chemistry—TRIGA site, Johannes Gutenberg—University of Mainz, Germany*

**Material and Methods**

**Reagents and instrumentation**

All reagents were of the best grade available and were purchased from common suppliers. All culture reagents were from Gibco BRL, Life Technologies (Grand Island, NY). The cancer associated prostate fibroblast cell line (hTERT PF179T, ATCC CRL-3290, Lot Number 0303) and the human glioblastoma cell line (U87MG, ATCC HTB-14, Lot number 4095) were acquired from American Type Culture Collection (ATCC, Manassas, Virginia, USA). The human prostate adenocarcinoma cell line PC3 (CLS-300312, Lot number 816SF) was obtained from Cell Lines Service GmbH (CLS, Eppelheim, Germany). The human serum was commercially available by Sigma (H4522). The GalliaPharm® Ge-68/Ga-68 generator was available from Eckert & Ziegler (Berlin, Germany). The quality control of the precursor and the radiotracer was performed by an analytical Reverse-Phase High Performance Liquid Chromatography (RP-HPLC) on an analytical Nucleosil 120-5 column C18 (250 x 4.5 mm) applying a linear gradient of 15-90% solvent B in 30 min at a flow rate of 1 mL/min. (solvent A, 0.1% trifluoroacetic acid (TFA)/water (H_2_O); solvent B, 0.1%TFA/acetonitrile (ACN). Ultraviolet detection was performed using a Knauer detector at 214 nm. For radioactivity measurement, a Na(TI) well-type scintillation Gina star was used. The radiotracer solutions for the experiments were prepared by dilution with 0.9% NaCl (Bichsel AG, Interlaken, Switzerland). ESI-MS mass spectra were acquired on a Bruker Daltonics Esquire 3000 plus device.

Quantitative γ-counting was performed with a COBRA II γ-system well counter from Packard Instrument (USA). For μPET/CT studies, a dedicated micro-PET/SPECT/CT scanner (Albira Si; Bruker Biospin, Ettlingen, Germany) was used. All experiments were carried out two times in triplicate.

Mice were purchased from Charles River Laboratories (Domain des Oncins, France), pentobarbital natrium (150mg/kg) from Streuli Pharma SA (Uznach, Switzerland).

**Radiochemistry/ Quality control of the radiotracer**

[^68^Ga]Ga-DATA^5m.^SA.FAPi was prepared within 5 min, using the Modular-Lab PharmTracer module by Eckert & Ziegler (Berlin, Germany). The radiolabeling performance of DATA^5m^.SA.FAPi was assessed at pH 4.0 (0.2 M sodium acetate buffer), at room temperature (RT) using the minimum conjugate amount. Briefly, the Ge-68/Ga-68 generator was eluted with 5 mL HCl 0.1 N and the eluate (~300 MBq) was loaded onto a cation exchange column (Strata-XC, Phenomenex). Gallium-68 was eluted with 700 μL of a mixture of 5.5 M NaCl / 0.1 M HCl directly in a vial containing 400 μL of 1.8 M sodium acetate buffer (pH 4.3), 2 mL H_2_O, 200 μL of EtOH, and 20 μg (approximately 20 nmol) of the conjugate, followed by SepPak C-18 purification to remove uncomplexed gallium-68. The purified [^68^Ga]Ga-DATA^5m.^SA.FAPi was eluted in 2.7 mL of a solution of 12% EtOH in PBS.

To prepare 5.5M NaCl/ 0.1 M HCl; 0.530 mL of HCl 30 % (Ultrapure) is added to 49.5 mL of 5.5M NaCl (total volume 50 mL).

After the labelling with gallium-68 and the quality control of [^68^Ga]Ga-DATA^5m^.SA.FAPi, one equivalent of ^nat^Ga(NO_3_)_3_ (1.1 µmol/mL) was added to the radiolabelling solution. The final solution was incubated at RT for 10 min to obtain structurally characterized homogeneous ligand which were used for the saturation binding studies. The homogeneity was determined by HPLC, showing one peak.

The quality control of [^68^Ga]Ga-DATA^5m^.SA.FAPi was performed by radio-HPLC as described in the reagents and instrumentation. The presence of free gallium-68 and ^68^Ga-labelled colloid in the ^68^Ga-labelled DATA^5m^.SA.FAP**i** preparation was quantified by radio thin layer chromatography (Radio-TLC) using ITLC-SG-plates (Glass microfiber chromatography paper impregnated with silica gel) and two different mobile phase eluents: a) 0.1 M Na-citrate; b) MeOH / 1 M ammonium acetate (1 / 1, v / v).

**Lipophilicity / Protein binding studies and metabolic stability in human serum**

The lipophilicity (LogD_Octanol/PBS_, pH 7.4) was estimated by the “shake-flask” method: The labelled conjugate (100 pmol; 1.6 MBq) was added to a 1:1 mixture of 1-octanol (500 μL) and PBS (500 μL, pH 7.4). The mixture was intermittently vortexed for 1 h to reach the equilibrium and then centrifuged (3000 rpm) for 10 min. From each phase, an aliquot (50 μL) was pipetted out and measured in a gamma-counter. Each measurement was repeated five times. Care was taken to avoid cross-contamination between the phases. The partition coefficient was calculated as the average log ratio of the radioactivity in the organic fraction and the PBS fraction.

^68^Ga-labelled DATA^5m^.SA.FAPi (100 pmol; 1.6 MBq) was incubated with commercially available human serum (0.5 mL) at 37°C for 30 min. When the incubating period was completed, proteins were precipitated with a solution of 1 mL MeOH/ACN (1:1). Centrifugation (10 min, 9660g) was performed for the separation of proteins. After careful separation of the two phases, the respective activities were measured in a gamma-counter, followed by determination of the percentage of the radiotracer which binds to the serum proteins.

A sample of the supernatant after filtration (0.22 μm Millex-GV filter, Merck Millipore Ltd, Tullagreen, IRL) was monitored by HP-HPLC to verify the presence of potential metabolites.

**Cell lines**

The human prostate adenocarcinoma cell line PC3 was cultured in Dulbecco's Modified Eagle Medium (DMEM) with low glucose (1g/l): F-12 Nutrient Mix with GlutaMAX^™^-I (1:1 mixture ratio). The human glioblastoma cell line U87MG was cultured in Dulbecco's Modified Eagle Medium (DMEM) with low glucose (1g/L), supplemented with NEAA 1% and sodium pyruvate 1mM. The cancer associated prostate fibroblast cell line CAF was cultured in the same conditions with Eagle's Minimal Essential Medium (EMEM) supplemented with sodium bicarbonate 1500 mg/L and puromycin 1 μg/mL. In each case, the medium was supplemented with 10% fetal bovine serum (FBS), penicillin (100 U/mL) and streptomycin (100 µg/mL). All the cell lines were cultured at 37°C and 5% CO_2_.

**Western blot analysis**

FAP protein level for PC3, U87MG, and human CAF cells were assessed by Western blotting. After incubation, cells were lysed in RIPA buffer (ThermoFisher) containing protease inhibitors (Roche). Total protein concentration was determined with the BCA™ protein assay kit (Pierce). Samples were loaded into a pre-cast NuPAGE 4-12%, Bis-Tris, (1.0 mm, 10 wells) (ThermoFisher) for electrophoresis, and transferred to Polyvinylidene difluoride (PVDF) membranes (ThermoFisher). Subsequently, membranes were blocked with 5% non-fat milk at room temperature, probed with the primary antibodies overnight at 4°C, and incubated with a secondary antibody solution for one hour at room temperature. Membranes were then visualized with Li-Cor system (Li-Cor Odyssey^®^ system vs 2.1 (LI-COR Biosciences,Lincoln, US)) and associated software (CLX Image Studio v. 5.2 (LI-COR Biosciences, Lincoln, US)). The antibodies employed rabbit monoclonal anti-FAP (1/1000, ab207178, Abcam. Cambridge, UK) and rabbit monoclonal anti-GAPDH (1/10000, ab181602, Abcam. Cambridge, UK), goat anti-rabbit IgG (H&L) IRDye 800CW (1/5000-1/15000, 926-32211, LI-COR Biosciences. Lincoln, US). GAPDH was used as an internal control.

**Radioligand binding assay**

For radioligand binding assays, the PC3, U87MG and CAF cells were seeded at a density of 0.8-1 million cells per well in 6-well plates. In case of the CAF cells the plates were pre-coated with BME (Cultrex Reduced Growth Factor Basement Membrane Extract, RGF BME, R&D System, Minneapolis, USA) to enhance cell attachment. The cells were incubated overnight with their cultivation medium. The next day, the medium was removed and the cells were incubated for 30 min at 37 °C, with 0.8 mL of fresh medium. Afterwards, the plates were placed on ice for 30 min, followed by addition of approximately 2.5 pmol of ^68/nat^Ga-labelled DATA^5m^.SA.FAPi (100 µL) and PBS pH 7.4 (100 µL), resulting in a total volume of 1 mL per well. After the addition of the radioligand, the cells were incubated for 120 min at 4 °C. Non-specific binding was determined in the presence of UAMC1110 at a final concentration of 1 μM. After the completion of the incubation, the cells were washed twice with ice-cold PBS, followed by solubilization with 1 N NaOH. The cell-associated radioactivity was measured using a gamma-counter. For all the cell studies the values are normalized for 1x10^6^ cells per well and all data are from two independent experiments with triplicates in each experiment.

**Saturation binding studies**

For receptor saturation analysis, the CAF cell line overexpressing FAP were seeded at a density of 0.8-1 million cells per well in 6-well plates, pre-coated with BME (Cultrex Reduced Growth Factor Basement Membrane Extract, RGF BME, R&D System, Minneapolis, USA) to enhance cell attachment. The cells were incubated overnight with medium (EMEM supplemented and containing 10% FBS, 100 U/mL penicillin and 100 μg/mL streptomycin). The next day, the medium was removed and the cells were incubated for 30 min at 37 °C, with 0.8 mL of fresh medium. Afterwards, the plates were placed on ice for 30 min followed by incubation with increasing concentrations of ^68/nat^Ga-labelled DATA^5m^.SA.FAPi (0.1-10 nM final concentration in the wells; 100 µL of radioligand solution and 100 µL of PBS pH 7.4 were added to the cells with 0.8 mL medium in the well). After the addition of the radioligand, the cells were incubated for 120 min at 4 °C. Non-specific binding was determined in the presence of UAMC1110 at a final concentration of 1 μM. After the completion of the incubation, the cells were washed twice with ice-cold PBS, followed by solubilization with 1 N NaOH. The cell-associated radioactivity was measured using a gamma-counter. Specific binding was plotted against the total molar concentration of the added radiotracer. The K_d_ values and the concentration of the radiotracer required to saturate the receptors (B_max_) were determined by nonlinear regression using GraphPad (Prism 8 Graph Pad Software, San Diego, CA). For all the cell studies the values are normalized for 1x10^6^ cells per well and all data are from two independent experiments with triplicates in each experiment.

**Internalization studies**

For internalization experiments, CAF cells were seeded into 6-well plates pre-coated with BME and treated as described at the saturation binding studies. On the day of the experiment, approximately 2.5 pmol (100 µL) of the radiotracer was added to the medium (total volume 1.5 mL) and the cells were incubated (in triplicates) for 15, 30, 60, 90, 120, 180 and 240 min at 37 °C, 5% CO_2_. To determine nonspecific membrane binding and internalization, excess of UAMC1110 (final concentration 1 μΜ) was added to selected wells. At each time point, the internalization was stopped by putting the plates on ice, removing the medium and washing the cells twice with ice-cold PBS. To remove the receptor-bound radioligand, an acid wash was carried out twice with a 0.1 M glycine buffer pH 2.8 for 5 min on ice. Finally, cells were solubilized with 1 N NaOH. The radioactivity of the culture medium, the receptor-bound, and the internalized fractions were measured in a γ-counter.

**Biodistribution Studies**

Ten pmol (0.06-0.09 MBq) of [^68^Ga]Ga-DATA^5m^.SA.FAPi in 100 µL of NaCl 0.9 % were injected intravenously into the tail vein of U87MG or PC3 tumor bearing mice. The PC3 tumor bearing mice were used between the 15^th^ and 17^th^ day after the implantation and the size was 281 ± 75 mm^3^. The U87MG tumor bearing mice were used between the 7^th^ and 9^th^ day after the implantation and the size was 210 ± 34 mm^3^. Animals were terminally anesthetized by intraperitoneal injection of an overdose of pentobarbital natricum (150mg/kg; Streuli Pharma SA, Uznach, Switzerland) at 1, 2 and 3 h after injection of the ^68^Ga-labelled DATA^5m^.SA.FAPi.

The organs of interest were dissected and weighted, and the radioactivity in tissue samples was counted in a γ-counter. Biodistribution data are given as percent of injected activity per gram of tissue (% IA/g) and are means ± SD (n = 4). To demonstrate the specificity of binding, U87MG or PC3 xenografts were co-injected with 10 pmol of [^68^Ga]Ga-DATA^5m^.SA.FAPi along with 20 nmol of UAMC1110 (total injected volume: 100 µL) and biodistribution studies were performed 2 h after injection.

After the biodistribution studies the tumors were checked for their vascularization and if they possess blood pool or if they are necrotic. Both tumor types were “clean” without extended vascularization, there was not blood pool in the center of the tumors and we also did not observe any necrotic signs.

**Small-Animal PET/CT Studies**

PET images were obtained upon injection of 200 pmol of [^68^Ga]Ga-DATA^5m^.SA.FAPi (1.2-1.5 MBq/100 μL) on U87MG or PC3 xenografts at 1, 2 and 3 h after injection. Static imaging was acquired for a time period between 10 and 45 min at 1, 2 and 3 h post injection, respectively. To visualize the extent of FAP-specific tumor uptake, PET/CT blocking studies were performed as described above, and static scans were obtained for 30 min 2 h after the injection of the radiotracer along with the blocking agent (20 nmol).

PET images were corrected for gallium-68 decay and reconstructed with 12 iterations of maximum a posteriori (MAP) algorithm using a voxel size of 0.25 mm. No correction was applied for attenuation. In addition, a point spread function iterative deconvolution was applied. The images were normalized, filtered using a Gaussian 3D algorithm with a 1.3 mm isotropic kernel and generated using PMOD software. The CT was carried out using step and shoot mode and employed 45 kVp and 400 uA as settings. The images were reconstructed using filtered back projection algorithm. The color scale was set from 0 to 25 % IA/g to allow for qualitative comparison among the images.

**Clinical assessment of [^68^Ga]Ga-DATA^5m^.SA.FAPi**

**Study design**

Imaging with [^68^Ga]Ga-DATA^5m^.SA.FAPi was performed in the context of potential individual treatments with a therapeutic FAPi ligand to identify sufficient expression of the molecular target in patients with metastatic castration resistant prostate cancer (mCRPC) without further treatment option. Eligible patients had a proven mCRPC, completed treatment options according to current clinical practice (including [^177^Lu]Lu-PSMA-617 therapy), no clinically significant concomitant diseases (e .g. renal failure, liver dysfunction or cardiovascular disease), and an adequate performance status (ECOG < 2). All patients gave written informed consent to undergo [^68^Ga]Ga-DATA^5m^.SA.FAPi PET/CT. The retrospective analysis of the dosimetric results was presented to the ethics committee of the Rostock University Medical Center, which waived the need for a formal review. The anonymized analyses were carried out in accordance with the declaration of Helsinki and its later amendments and the legal considerations of clinical guidelines.

**Patients**

Six male patients with mCRPC were included in this dosimetric study (mean age 72.2 ± 7.6 y, mean weight 80.3 ± 11.3 kg and mean height 177.8 ± 8.1 cm). The mean activity administered was 167.7 ± 54.9 MBq (range 136.5 ... 261.0 MBq) of ^68^Ga-DATA^5m^.SA.FAPi, depending on both patient weight and synthesis yield due to ^68^Ge/^68^Ga generator aging. Table 1S summarizes the patient characteristics.

**Table 1S.** Characteristics of the patients who underwent [^68^Ga]Ga-SA-FAPi-PET/CT for potential FAPi-based therapy.

| Pat No | Age [yr] | Height [cm] | Weight [kg] | Pretreatments | Metastases | Activity [MBq] |
| --- | --- | --- | --- | --- | --- | --- |
| 1 | 63 | 194 | 78 | DXL, ABI, ENZA, [^177^Lu]Lu-PSMA-617 | OSS, LN | 225 |
| 2 | 67 | 170 | 73 | DXL, CXL, [^177^Lu]Lu-PSMA-617 | OSS, LN, VIS | 261 |
| 3 | 80 | 176 | 73 | DXL, CXL, ABI, ENZA, [177Lu]Lu-PSMA-617 | OSS, LN | 137 |
| 4 | 78 | 180 | 83 | DXL, CXL, ABI, MITO, ENZA, [^177^Lu]Lu-PSMA-617 | OSS, LN | 136 |
| 5 | 81 | 170 | 71 | DXL, CXL, ABI, ENZA, [^177^Lu]Lu-PSMA-617 | OSS, LN, VIS | 113 |
| 6 | 64 | 177 | 104 | DXL, ABI, ENZA, [^177^Lu]Lu-PSMA-617 | OSS, LN | 134 |
|  |  |  |  |  |  |  |
| Mean | 72,2 | 177,8 | 80,3 |  |  | 167,7 |
| SD | 7,6 | 8,1 | 11,3 |  |  | 54,9 |
| Median | 72,5 | 176,5 | 75,5 |  |  | 136,5 |

DXL: Docetaxel; CXL; Cabacitaxel; ABI: Abiraterone; ENZA: Enzalutamide, MITO; Mitoxantrone; OSS – osseous; HEP – hepatic; VIS – visceral; LN – lymph nodes

**PET/CT imaging**

Overall, seven PET images from the base of the skull to the mid-thigh were acquired using a Gemini TF 16 PET/CT (Philips Healthcare) including whole-body CT imaging, performed as auxiliary CT (120 kVp, 30 mAs). All patients were asked to void the urinary bladder before starting the acquisition. The first 6 PETs were taken within the first 1h, starting with the injection and covering a time span of 10 min each. Prior to this series the corresponding auxiliary CT was acquired. Patients were instructed to void the urinary bladder after the 1 h scan period. The seventh PET was started after 3 to 3.5 hours, with an acquisition time of 2.5 min per bed. For this series, a separate auxiliary CT was acquired with the above acquisition parameters. The PET data were reconstructed using the manufacture’s standard whole body BLOB-OS-TF reconstruction protocol (3 iterations, 31 subsets), corrected for randoms, scatter, and decay and the auxiliary CT was used for attenuation correction.

**Dosimetry – calculation of the absorbed doses**

The mean absorbed doses (AD) were calculated according to the MIRD scheme [1]. Co-registration of PET/CT datasets of all time points, organ delineation, and the determination of the activity accumulation were performed using Hermes Hybrid Viewer 6.13 (Hermes Medical Solutions, Sweden). Dose calculation was performed using OLINDA, version 2.2.3 (Hermes Medical Solutions, Sweden). Volumes of interest (VOI) were semi-automatically drawn on the axial slices of the CT of each PET/CT study for the following body regions: lungs, heart content, liver, spleen, stomach content, kidneys. The activity accumulated in each source organ was determined for each time point by multiplying the mean activity concentration by the organ volume and then normalized to the administered activity. Time-activity curves (TAC) for all source organs were determined using an in-house programmed fitting program (LabVIEW, National Instruments) based on the Levenberg-Marquardt algorithm assuming bi-exponential fitting to the data points. The Time-Integrated-Activity-Coefficients TIAC were calculated by subsequent integration to infinity, conservatively assuming a physical decay after the last measurement point. The rest of the body-TIAC was calculated by subtracting the sum of the TIAC of the source organs from the whole-body TIAC. Bone marrow dosimetry was estimated by drawing three red marrow VOIs, in the head of humeral bone, in the heads of the femoral bone and in the lumbar vertebrae L3-L4, respectively using the red marrow mass of the ICRP-89 adult male reference phantom [2]. All calculated TIAC were transferred as input into OLINDA and the organ AD and effective dose (E) were calculated, using the voxel-based phantoms including the standardized organ masses according to ICRP-89 and the ICRP103 tissue weighting factors. Since no urine samples were collected, the activity excreted at 1 h was estimated based on the bladder activity of the 6th PET/CT, assuming for simplicity that the radioactivity was almost completely excreted immediately after this PET acquisition. The corresponding TIAC for the urinary bladder was calculated from the excreted activity divided by the applied activity, serving as input for the dynamic bladder model implemented in OLINDA assuming a bladder voiding interval of 1 h.

**Binding stability in tumors**

Tumors (bone and soft tissue) with increased FAPi uptake were contoured on the PET images with a standard threshold of 40 % of the respective SUV_max_ with subsequent manual adjustment if necessary. To assess the binding stability in the tumor tissue, the tumor-to-background ratio (TBR) was calculated as the quotient of the SUV mean values of the tumor and the background VOI. For this purpose, 3 VOI (diameter 3 cm) were placed in close proximity to each contoured tumor lesion in areas of non-specific tracer uptake.

**Results**

**Quality control of the radiotracer/Stability**

Based on the amount of the precursor which was used for the radiolabeling and assuming we lose about 20% during the labeling, the apparent molar activity (A_m_) was ranging between 15-22 GBq/µmol (no decay corrected).

DATA^5m^.SA.FAPi was successfully labelled with gallium-68 at RT in >98% radiochemical purity as determined by analytical RP-HPLC (Fig. 1Sa). Radiotracer stability over time was also determined and neither radiolysis or decomposition was observed for a period of 4 hour post labeling.

With regard to the detection of the formation of colloids by ITLC, using the first radio-TLC eluent, [^68^Ga]Ga-DATA^5m^.SA.FAPi and ^68^Ga-labelled colloid remain immobilized at the starting point, whereas free gallium-68 moves with the mobile phase. When the second eluent is used, only the [^68^Ga]Ga-DATA^5m^.SA.FAPi moves with the mobile phase / solvent front.

The metabolic stability of [^68^Ga]Ga-DATA^5m^.SA.FAPi in human serum was monitored by RP-HPLC. Apart from minute amounts of a polar metabolite (<0.5%) no change in the chromatogram pattern was observed after 30 min of incubation at 37 °C (Fig. 1Sb)


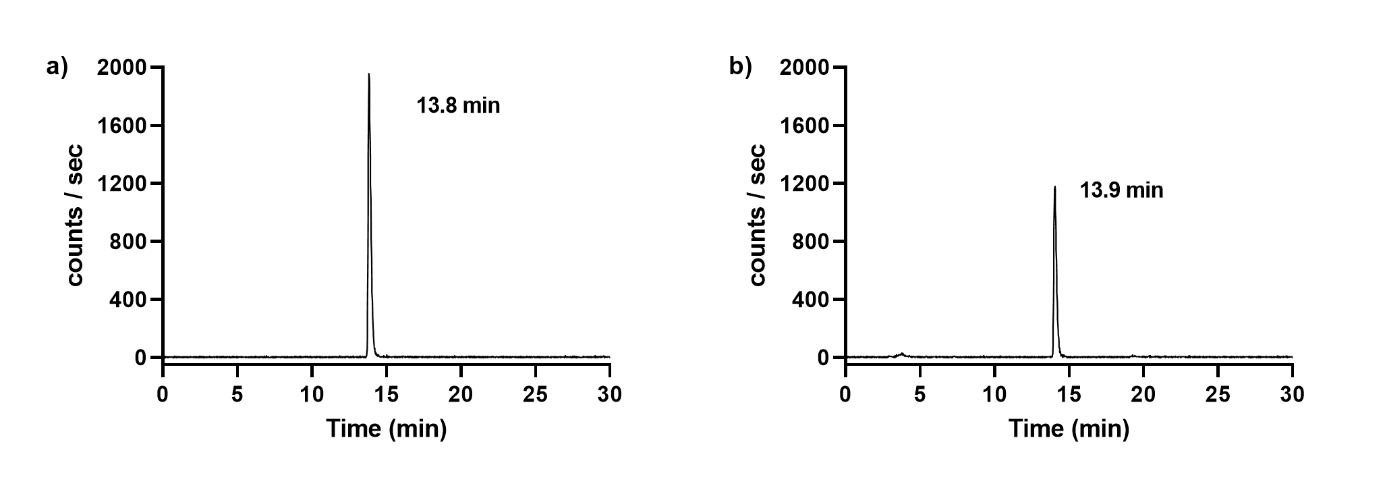
**Fig. 1S** **a** HPLC profiles of [^68^Ga]Ga-DATA^5m^.SA.FAPi, **b** HPLC profile of [^68^Ga]Ga-DATA^5m^.SA.FAPi after 30 min of incubation in human serum.

**Saturation Binding Studies**

**Fig.** **2S** Saturation binding study on CAF cells, using increasing concentrations of ^68/nat^Ga-DATA^5m^.SA.FAPi (0.1 to 10 nM). Total, non-specific and specific binding are displayed.

**Clinical assessment of [^68^Ga]Ga-DATA^5m^.SA.FAPi**


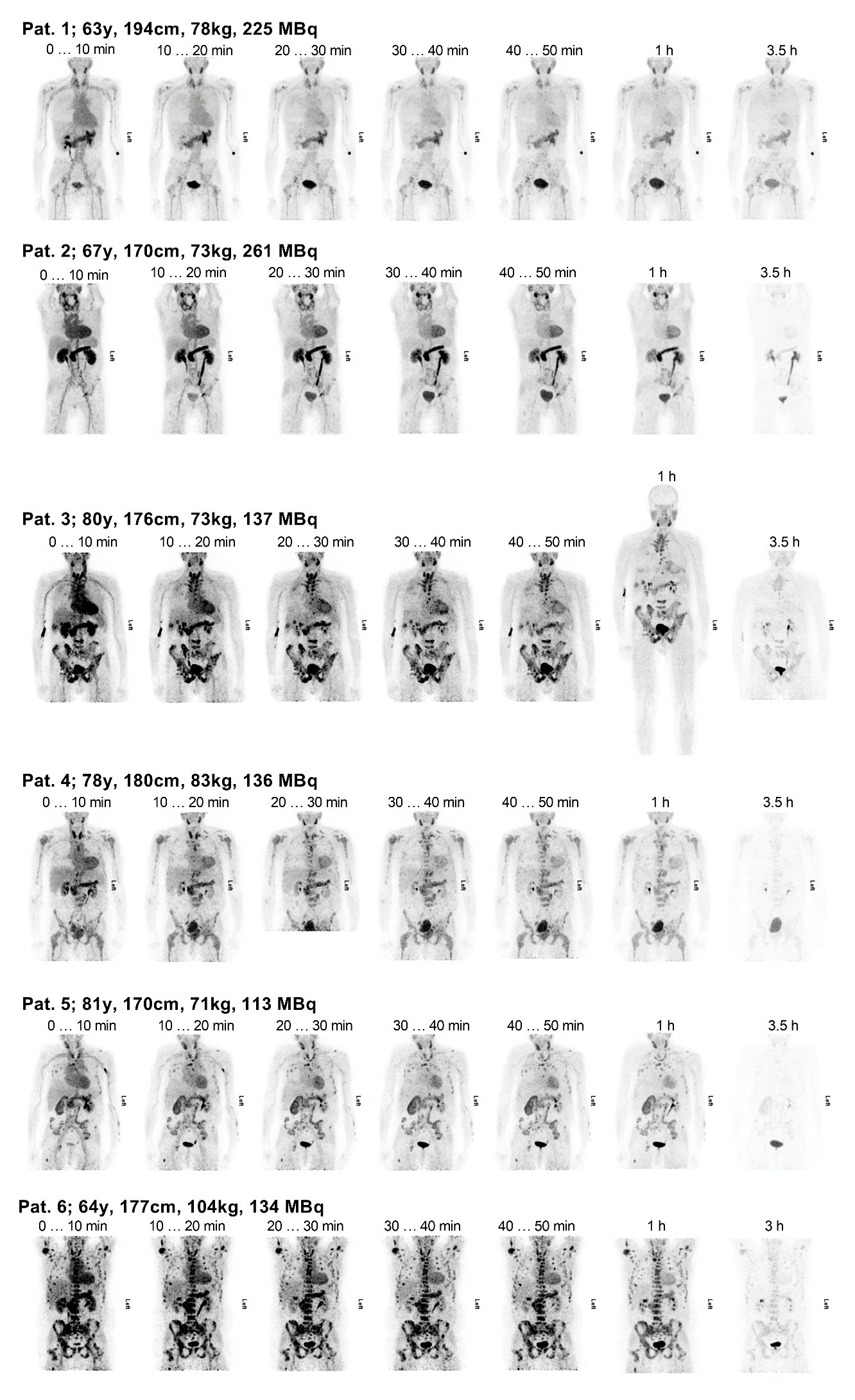


**Fig. 3S.** An overview of the MIP for all patients and all time points.


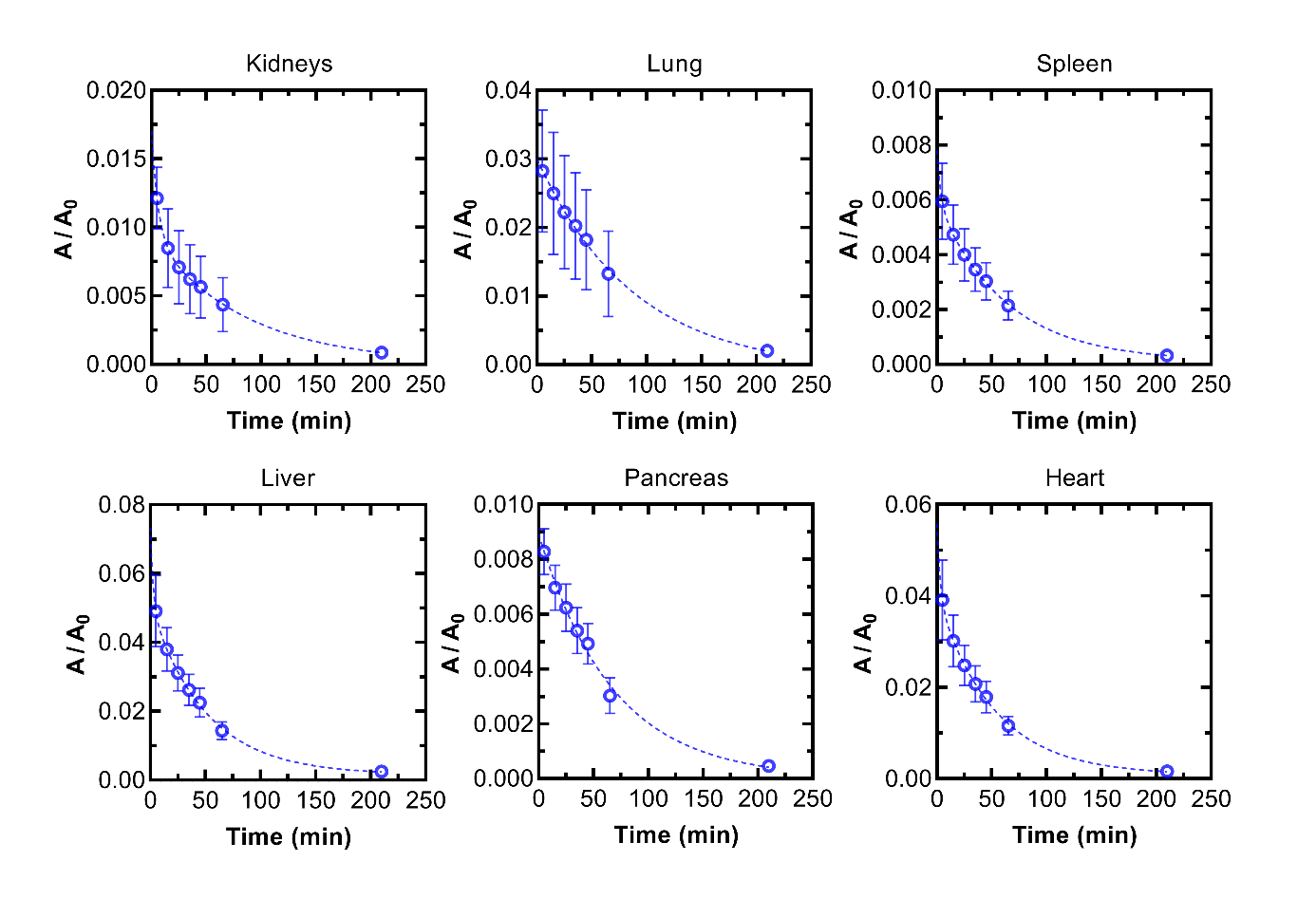


**Fig 4S.** Detailed graphic depicting time activity curve for each source organ

**Table 2S** Average organ AD and ED (tissue weighting factors according to ICRP-103) for a 1h urinary voiding interval. Organ masses of the adult male reference phantom according to ICRP-89 as implemented in OLINDA 2.2.3 were used.

| Organ AD [mGy/MBq] |  |  |  |  |  |  |  |  |
| --- | --- | --- | --- | --- | --- | --- | --- | --- |
| **Target organ / patient** | #1 | #2 | #3 | #4 | #5 | #6 | Mean | SD |
| Adrenals | 1.22E-02 | 1.42E-02 | 1.30E-02 | 1.32E-02 | 1.58E-02 | 1.11E-02 | 1.33E-02 | 1.48E-03 |
| Brain | 7.79E-03 | 7.66E-03 | 8.83E-03 | 8.51E-03 | 8.37E-03 | 8.25E-03 | 8.24E-03 | 4.03E-04 |
| Esophagus | 1.09E-02 | 9.96E-03 | 1.11E-02 | 1.06E-02 | 1.10E-02 | 9.52E-03 | 1.05E-02 | 5.82E-04 |
| Eyes | 7.79E-03 | 7.67E-03 | 8.83E-03 | 8.51E-03 | 8.37E-03 | 8.25E-03 | 8.24E-03 | 4.01E-04 |
| Gallbladder Wall | 1.04E-02 | 1.06E-02 | 1.14E-02 | 1.17E-02 | 1.14E-02 | 1.04E-02 | 1.10E-02 | 5.30E-04 |
| Left colon | 9.91E-03 | 9.91E-03 | 1.08E-02 | 1.05E-02 | 1.07E-02 | 1.00E-02 | 1.03E-02 | 3.75E-04 |
| Small Intestine | 9.58E-03 | 9.56E-03 | 1.07E-02 | 1.04E-02 | 1.04E-02 | 9.94E-03 | 1.01E-02 | 4.33E-04 |
| Stomach Wall | 1.34E-02 | 1.48E-02 | 1.41E-02 | 1.31E-02 | 1.58E-02 | 1.17E-02 | 1.38E-02 | 1.30E-03 |
| Right colon | 9.50E-03 | 9.52E-03 | 1.06E-02 | 1.04E-02 | 1.03E-02 | 9.81E-03 | 1.00E-02 | 4.33E-04 |
| Rectum | 9.06E-03 | 8.99E-03 | 1.03E-02 | 9.93E-03 | 9.80E-03 | 9.61E-03 | 9.62E-03 | 4.66E-04 |
| Heart Wall | 2.51E-02 | 2.90E-02 | 2.82E-02 | 2.57E-02 | 3.03E-02 | 1.97E-02 | 2.63E-02 | 3.47E-03 |
| Kidneys | 2.44E-03 | 8.31E-03 | 3.48E-03 | 3.32E-03 | 6.41E-02 | 1.96E-03 | 1.39E-02 | 2.25E-02 |
| Liver | 1.20E-02 | 1.76E-02 | 1.67E-02 | 1.48E-02 | 1.52E-02 | 1.02E-02 | 1.44E-02 | 2.57E-03 |
| Lungs | 5.34E-02 | 2.39E-02 | 2.98E-02 | 2.50E-02 | 3.20E-02 | 1.43E-02 | 2.97E-02 | 1.20E-02 |
| Pancreas | 4.45E-02 | 7.06E-02 | 3.06E-02 | 3.92E-02 | 3.34E-02 | 3.24E-02 | 4.18E-02 | 1.37E-02 |
| Prostate | 9.03E-03 | 8.97E-03 | 1.02E-02 | 9.89E-03 | 9.79E-03 | 9.56E-03 | 9.57E-03 | 4.47E-04 |
| Salivary Glands | 8.49E-03 | 8.31E-03 | 9.57E-03 | 9.21E-03 | 9.08E-03 | 8.91E-03 | 8.93E-03 | 4.26E-04 |
| Red Marrow | 7.70E-03 | 7.31E-03 | 8.28E-03 | 7.97E-03 | 8.03E-03 | 7.49E-03 | 7.80E-03 | 3.31E-04 |
| Osteogenic Cells | 6.89E-03 | 6.69E-03 | 7.60E-03 | 7.33E-03 | 7.32E-03 | 6.99E-03 | 7.14E-03 | 3.08E-04 |
| Spleen | 1.40E-02 | 2.10E-02 | 2.34E-02 | 1.39E-02 | 2.29E-02 | 9.63E-03 | 1.75E-02 | 5.22E-03 |
| Testes | 8.12E-03 | 8.03E-03 | 9.24E-03 | 8.91E-03 | 8.76E-03 | 8.65E-03 | 8.62E-03 | 4.26E-04 |
| Thymus | 1.07E-02 | 9.96E-03 | 1.12E-02 | 1.06E-02 | 1.10E-02 | 9.56E-03 | 1.05E-02 | 5.72E-04 |
| Thyroid | 9.78E-03 | 8.87E-03 | 1.02E-02 | 9.74E-03 | 9.83E-03 | 9.11E-03 | 9.59E-03 | 4.54E-04 |
| Urinary Bladder Wall | 5.00E-02 | 6.06E-02 | 3.84E-02 | 3.84E-02 | 3.84E-02 | 7.77E-02 | 5.06E-02 | 1.46E-02 |
| Total Body | 9.82E-03 | 9.42E-03 | 1.05E-02 | 1.01E-02 | 1.03E-02 | 9.38E-03 | 9.92E-03 | 4.21E-04 |
| **ED** (ICRP 103) [mSv/MBq] | 1.52E-02 | 1.28E-02 | 1.25E-02 | 1.39E-02 | 1.53E-02 | 1.12E-02 | 1.35E-02 | 1.48E-03 |

**Table 3S** Time-integrated activity coefficients (TIAC in units of MBq h/MBq) of the source organs considered for incorporation dosimetry.

| **Source organ / patient** | #1 | #2 | #3 | #4 | #5 | #6 | Mean | SD |
| --- | --- | --- | --- | --- | --- | --- | --- | --- |
| Stomach Contents | 2.40E-03 | 4.60E-03 | 3.10E-03 | 2.40E-03 | 4.70E-03 | 1.80E-03 | 3.32E-03 | 1.16E-03 |
| Heart Contents | 2.84E-02 | 3.91E-02 | 3.50E-02 | 3.08E-02 | 3.95E-02 | 2.09E-02 | 3.31E-02 | 6.85E-03 |
| Kidneys | 1.86E-02 | 3.50E-02 | 1.82E-02 | 1.90E-02 | 4.23E-02 | 9.80E-03 | 2.49E-02 | 1.19E-02 |
| Liver | 3.34E-02 | 4.44E-02 | 3.67E-02 | 5.36E-02 | 4.22E-02 | 3.41E-02 | 4.22E-02 | 6.79E-03 |
| Lungs | 1.45E-01 | 5.98E-02 | 7.64E-02 | 6.24E-02 | 8.23E-02 | 3.30E-02 | 6.28E-02 | 1.71E-02 |
| Pancreas | 1.03E-02 | 1.05E-02 | 6.40E-03 | 7.60E-43 | 9.20E-03 | 8.60E-03 | 6.94E-03 | 3.71E-03 |
| Spleen | 7.20E-03 | 8.00E-03 | 3.80E-03 | 5.20E-03 | 6.40E-03 | 4.50E-03 | 5.58E-03 | 1.48E-03 |
| Urinary Bladder Content | 3.58E-02 | 4.50E-02 | 2.48E-02 | 7.23E-02 | 6.31E-02 | 5.93E-02 | 5.29E-02 | 1.66E-02 |
| Rest of the body | 9.41E-01 | 9.30E-01 | 1.07E+00 | 1.03E+00 | 1.01E+00 | 1.0042 | 1.01E+00 | 4.63E-02 |

**References**

1. Bolch WE, Eckerman KF, Sgouros G, Thomas SR. MIRD pamphlet No. 21: a generalized schema for radiopharmaceutical dosimetry--standardization of nomenclature. J Nucl Med. 2009;50:477-84. doi:10.2967/jnumed.108.056036.
2. Basic anatomical and physiological data for use in radiological protection: reference values. A report of age- and gender-related differences in the anatomical and physiological characteristics of reference individuals. ICRP Publication 89. Ann ICRP. 2002;32:5-265.
